# Carbon black as an alternative cathode material for electrical energy recovery and transfer in a microbial battery

**DOI:** 10.1038/s41598-017-07174-z

**Published:** 2017-08-01

**Authors:** Xueqin Zhang, Kun Guo, Dongsheng Shen, Huajun Feng, Meizhen Wang, Yuyang Zhou, Yufeng Jia, Yuxiang Liang, Mengjiao Zhou

**Affiliations:** 1 Zhejiang Provincial Key Laboratory of Solid Waste Treatment and Recycling, School of Environmental Science and Engineering, Zhejiang Gongshang University, Hangzhou, 310012 China; 2 Advanced Water Management Centre, The University of Queensland, St Lucia, QLD 4072 Australia; 3 Center for Microbial Ecology and Technology, Ghent University, Coupure Links 653, B-9000 Ghent, Belgium

## Abstract

Rather than the conventional concept of viewing conductive carbon black (CB) to be chemically inert in microbial electrochemical cells (MECs), here we confirmed the redox activity of CB for its feasibility as an electron sink in the microbial battery (MB). Acting as the cathode of a MB, the solid-state CB electrode showed the highest electron capacity equivalent of 18.58 ± 0.46 C/g for the unsintered one and the lowest capacity of 2.29 ± 0.48 C/g for the one sintered under 100% N_2_ atmosphere. The capacity vibrations of CBs were strongly in coincidence with the abundances of C=O moiety caused by different pretreatments and it implied one plausible mechanism based on CB’s surface functionality for its electron capturing. Once subjected to electron saturation, CB could be completely regenerated by different strategies in terms of electrochemical discharging or donating electrons to biologically-catalyzed nitrate reduction. Surface characterization also revealed that CB’s regeneration fully depended on the reversible shift of C=O moiety, further confirming the functionality-based mechanism for CB’s feasibility as the role of MB’s cathode. Moreover, resilience tests demonstrated that CB cathode was robust for the multi-cycles charging-discharging operations. These results imply that CB is a promising alternative material for the solid-state cathode in MBs.

## Introduction

Nowadays increasing energy demand and adverse effects of energy extraction from irreversible fossil consumption on environment have catalyzed scientific focus on deriving energy from organic reservoirs (especially organic wastes e.g., domestic wastewater) via biotechnology. Among various microbial technologies, microbial fell cells (MFCs) offer an option for direct electricity harvest from organics by anodic microbial catalysis and have been used to recover electrical energy from domestic sewage^1, 2^. Originally consumable agents, such as potassium ferricyanide, potassium permanganate, were used in the cathode of MFCs as electron acceptors, while O_2_ was identified as an optimal alternative afterwards for practical applications as it is inexhaustible from ambient air^3^. However, O_2_ functioning as terminal acceptor has some disadvantages: (1) limited kinetics of the oxygen reduction especially when reaction occurs in solution that oxygen has low solubility thus low accessibility^4^; (2) high voltage losses at the cathode due to the high overpotential^1^; (3) charge losses resulted from aerobic metabolism as dissolved oxygen diffuses from the cathode compartment into the anode compartment^5^.

To eliminate the defects of oxygen-based MFCs, Xie *et al*.^6^ described a microbial battery (MB), a new type of microbial electrochemical cells (MECs), that was designed to use the solid-state cathode itself instead of O_2_ as electron acceptor. The MBs are superior to MFCs by improving energy recovery efficiency since charge loss can be prevented without O_2_ involvement and voltage loss can be reduced by the re-oxidation of electrode reaction rather than O_2_ reduction^6^. The operational concept of the solid-state cathode is like that of a rechargeable battery, which is technically dependent on the reversible capabilities of cathodic materials. Thus the configuration challenge for MBs is to find a right cathode material that is rechargeable and at the same time with the suitable redox-potential to ensure the microbial-mediated electron transfer from organic reservoirs into cathode; other significant considerations include robustness, cost-effectiveness, and energy-effective regeneration^6^. Based on these principles, a prussian blue cathode^7^ was further developed versus the originally-constructed silver-oxide electrode for a practical application implication, for the fact that silver is costly, and regeneration of silver oxidation is thermodynamically unfavorable and energy-consuming^6^. Enlightened by this, identification of more competing materials for solid-state cathode is quite desirable.

Carbon materials, such as carbon black (CB), activated carbon (AC), graphite, carbon cloth, carbon felt, and reticulated vitreous carbon, are the most commonly used electrode materials for MECs because of their low price, good biocompatibility and chemical stability^8, 9^. However, these properties have aroused a general illusion in the field of bioelectrochemistry that all carbon materials are chemically inert. For example, in several studie^10–12^, CB was fabricated into the air layer of the synthetic air-cathode in single-chamber MFCs, the role of which was viewed to be inert for gas diffusion rather than active catalysis. This illusion is also strengthened by the focus on the application of carbon materials (e.g., AC and CB) for contaminant remediation, and they are viewed as passive adsorbents^13–15^.

However, small portion of recent studies have broken this prominent belief and many carbon materials, including CB, AC, graphite, char, carbon nanotubes, and graphene oxide, were proved to be active in mediating specific oxidation and reduction reactions^16–18^. In the study by Robles *et al*.^19^, AC was used as a redox mediator for the enhanced degradation of azo dye in a MEC; Dong *et al*.^20^ first reported that AC functioned as a cheap oxygen reduction catalyst for efficient electricity generation in an air-cathode MFC; in a similar publication^21^, wood-derived carbon material was applied for an MFC anode to achieve satisfactory power density. Although the specific mechanisms for the active roles of carbon materials in these MECs were not fully understood, they actually have played a catalytic role to accelerate electron transfer rate. Some attributed these performance contributions to physical resolutions (such as high conductivity^22^ or high porousness^23^) of carbon materials), while some others attributed them to chemically active property of some carbon materials based on their redox-active surface functionalities^17^. Based on this indication, chemically-active carbon materials are potentially able to capture and store soluble electrons, and therefore play the role as solid-state electron donor.

Considering that the carbon material is one of the most promising nonprecious materials as it is both inexpensive and renewably accessible from waste biomass^23^, it may provide a more practical outlet for the application development in MBs. Therefore, in present work, we studied CB as a typical example of the carbon material to investigate the practicality of CB as the solid-state cathode of MBs. More specifically, we determined if CB can act as electron sink for biodegradable organic extraction and if its electron-capture ability can be regenerated by re-oxidation of CB. Besides, the functionality-based mechanism correlated to CB’s electron-transfer role was also elucidated based on the experimental results.

## Results and Discussion

### Electrochemical Redox Characterization of CB Cathode

**Figure 1:**
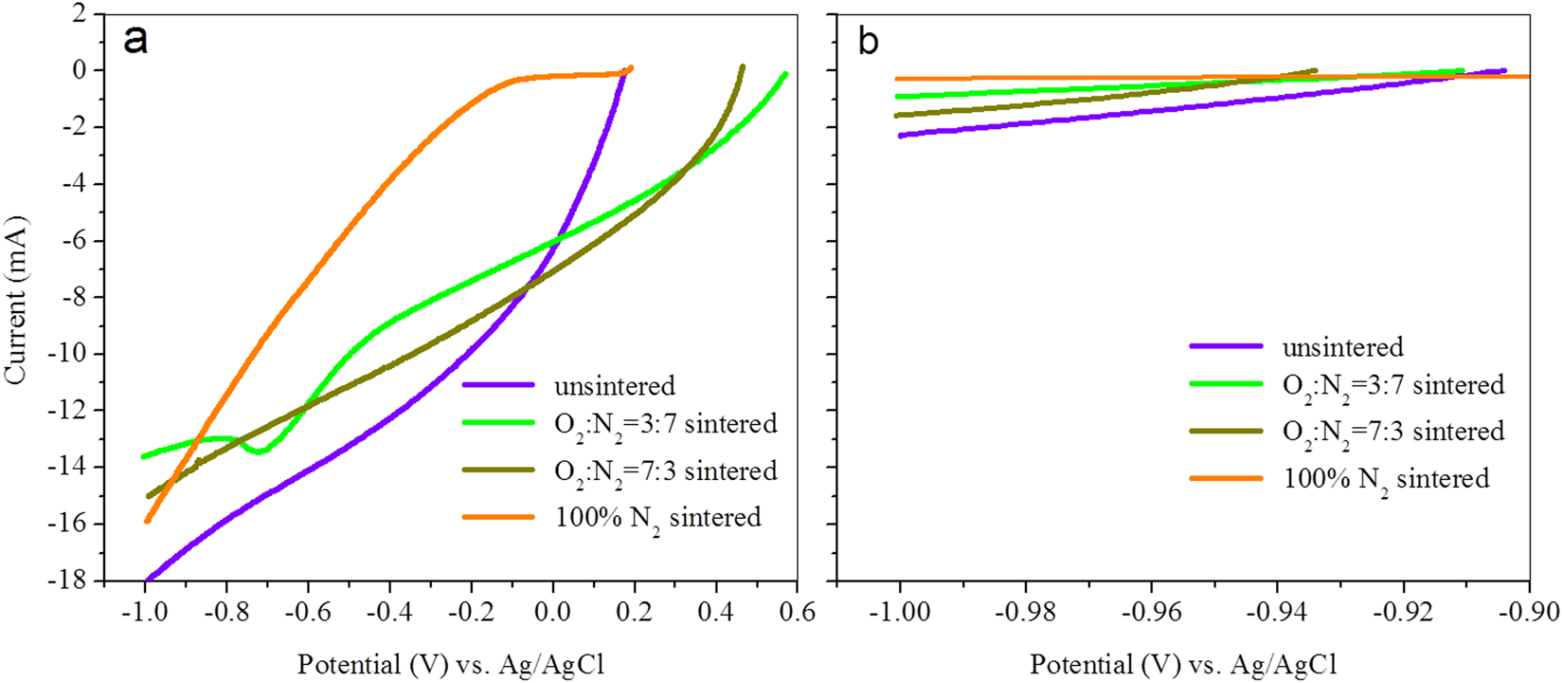
Linear sweep voltammetry (LSV) curves of CB electrodes (**a**: the curves of newly fabricated electrodes with different pre-treatments, **b**: corresponding curves of charged (fully saturated with electrons) electrodes).

The electrochemical redox properties of solid-state CB electrodes as characterized by LSVs are shown in Fig. 1. Curves exhibited active interactions on the interface between all four CB electrodes and electrolyte. As no hydrogen evolution was detected on the CB electrodes (the details of confirmation experiment and specific discussion can be found in Supplementary Information), it suggested the strong electron-capturing capability of CB electrodes. Similar to the results of LSVs, cyclic voltammetry curves (CVs) also showed obvious reduction peaks (Fig. S4a), rather than forming a rectangular shape of the voltammetry characteristics. It meant the electron-capturing capability of CB was from its redox-active property rather than double layer capacitance.

However, the open circuit potentials (OCPs) of four CB electrodes varied (Fig. 1a), which meant the different redox status of differently-treated CBs. We noted that the OCPs of electrodes were all positive than 0 V vs Ag/AgCl, and it implied the accessible turnover potential of CB’s electron-accepting characterization, which is a prerequisite for the selection of MB’s cathode material^6^. Theoretically, for a spontaneous electron transfer from anodic reservoirs to cathodes in MECs, a more positive oxidation potential compared with the anodic potential of −0.4 V (vs Ag/AgCl electrode) is compulsory for cathodic electron acceptors^2^. Thus, the potentials for electron-accepting turnover shown in the LSV curves meant that CB was capable of capturing electrons in MBs in terms of a microbial-mediated organic extraction.

**Figure 2:**
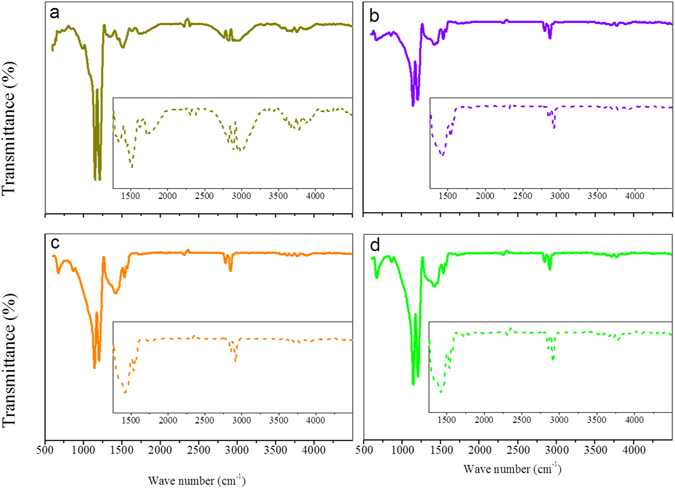
FT-IR profiles of CB electrodes (**a**: electrode unsintered, **b**: electrode sintered under 100%N_2_, **c**: electrode sintered under 30%O_2_ and 70%N_2_, **d**: electrodes sintered under 70%O_2_ and 30%N_2_).

Referring to surface functionalities of CBs, these findings were confirmed by FTIR analysis (Fig. 2 and Table S2). Readers are referred to the Supplementary Information for a more detailed discussion of these results. To summarize, (i) the notable vibration of C=C at 1440 cm^−1^ indicative of the existence of aromatic C^24, 25^ and the vibration of C=O at 1630 cm^−1^ indicative of a strong presence of quinone structures^25, 26^ both confirmed CB to be an excellent electron sink^27, 28^. (ii) The unsintered CB electrode wholly preserved the absorbance of the pristine CB with unsaturated C=C band and C=O, while only the quinone C=O band remained for the sintered electrodes. These results indicated that the functionality of the CB electrodes arose from the inherent nature of CB, and their activity varied according to the functional group shift.

**Figure 3:**
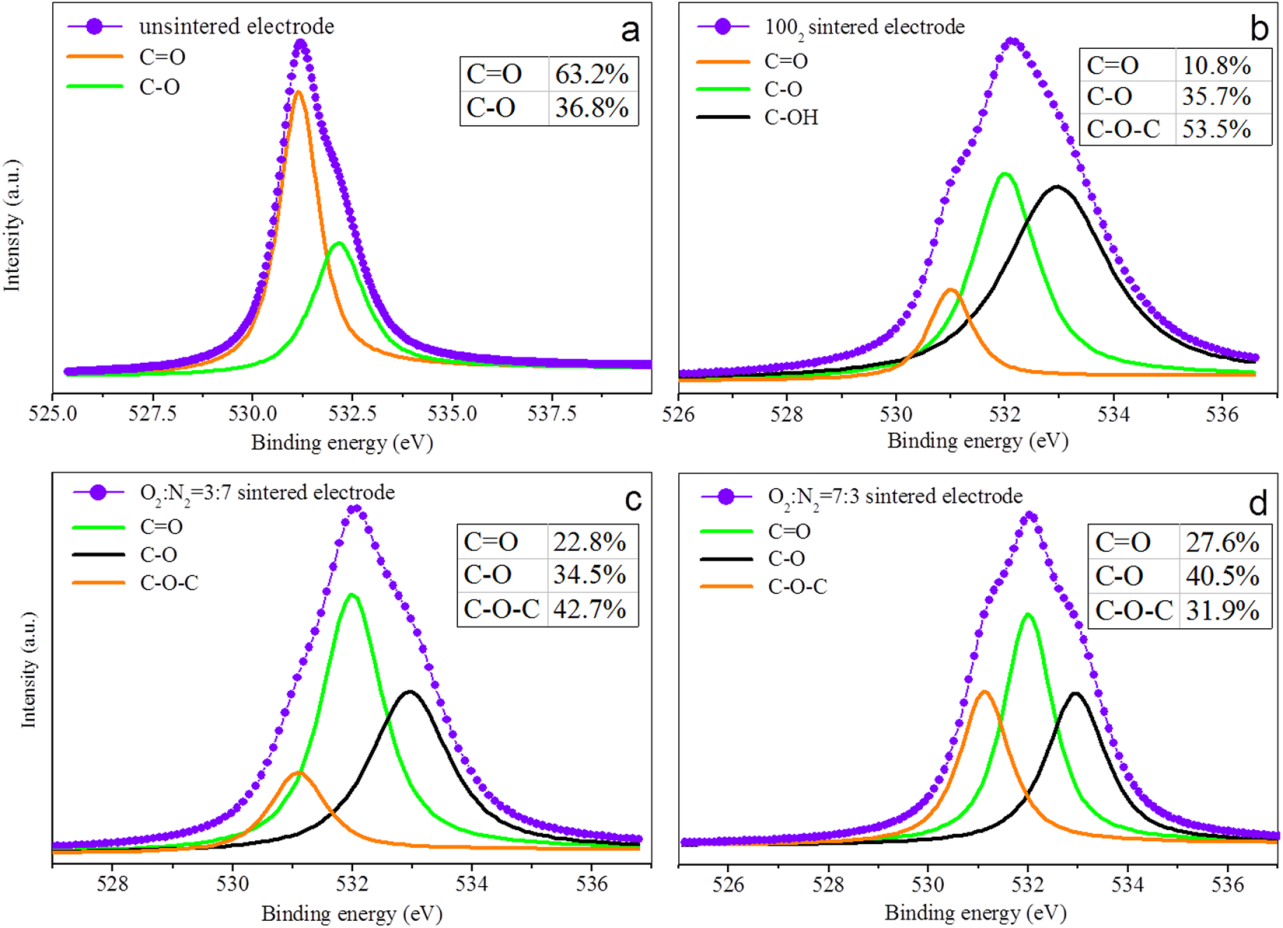
The high resolution O1s spectra of newly fabricated CB electrodes with different pre-treatments.

XPS spectra (Fig. 3 and Table S3; detailed discussion also in Supplementary Information) further demonstrated the existence of the quinone C=O moieties for the unsintered and sintered CBs^29, 30^. Previous studies have shown that the surface functionalities of carbon materials, including hydroxyl, carbonyl, carboxyl, and lactone moieties, can lead to reversible redox reactions, which can be expressed as follows^31, 32^:

1 $$ > {\rm{C}}\mbox{--}{\rm{OH}}\rightleftharpoons  > {\rm{C}}={\rm{O}}+{{\rm{H}}}^{+}+{{\rm{e}}}^{-}$$

2 $$\mbox{--}{\rm{COOH}}\rightleftharpoons \mbox{--}{\rm{COO}}+{{\rm{H}}}^{+}+{{\rm{e}}}^{-}$$

3 $$ > {\rm{C}}={\rm{O}}+{{\rm{e}}}^{-}\rightleftharpoons  > {\rm{C}}\mbox{--}{{\rm{O}}}^{-}$$

Thus the presence of C=O moieties, the indication of a strong electron-accepting capability of CB and no hydrogen evolution confirmed that the chemical activity of CB was closely related to its surface chemistry. Moreover, the C=O moiety abundance discrepancy (Fig. 3) of differently treated CB electrodes may explain their different redox status.

Notable changes were observed for the LSV curves of charged electrodes (Fig. 1b). The significantly varied current intensities all underwent a notable drop to a similar level, and the strong reductive tendencies disappeared. The same phenomenon was also observed for CVs (Fig. S4b). This ‘inert appearance’ arose from the electron saturation of the electrodes, was confirmed by decreases of the C=O moiety abundance for different electrodes to a similar percentage and other moieties providing similar proportion after electrodes being charged (Fig. S7). This proof was also in agreement with the final redox status of differently-treated CB in terms of the similar OCPs (around −0.9 V vs Ag/AgCl) (Fig. 1b). Meanwhile, the variation trend of OCP, i.e decreasing from different vales to the similar one, signified the conversion of CB by electron saturation and further proved the CB’s capability of electron-capturing. Further decreasing reduction potential resulted in hydrogen evolution on the surface of charged electrodes (the details discussion can be found in Supplementary Information) and it further proved CB’s chemical activity by no observation of hydrogen evolutions (with potential positive than −1.0 V) on the surface of uncharged electrodes (Fig. S2).

**Figure 4:**
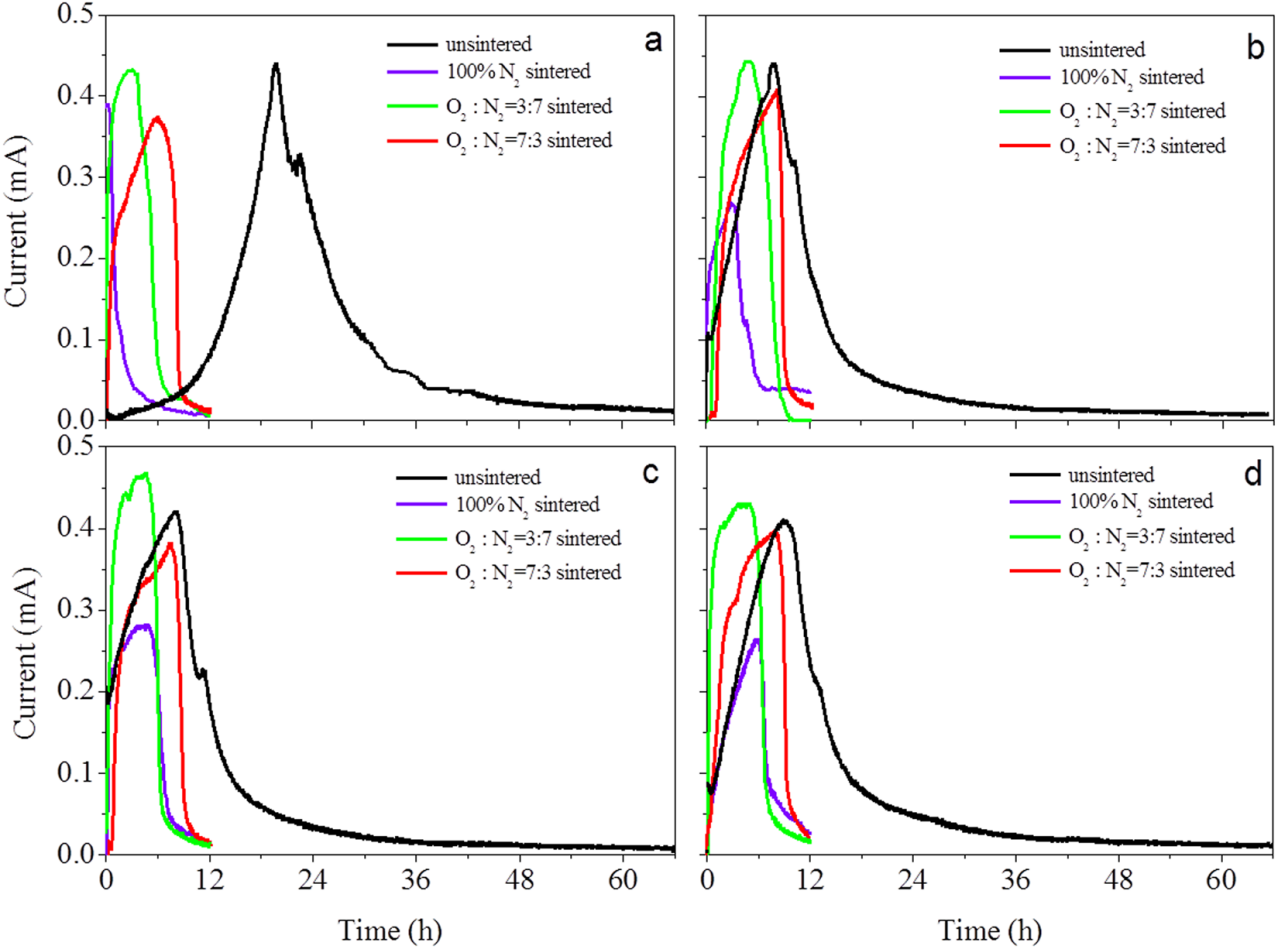
Charging curves of CB electrodes in MFCs (**a**: the newly fabricated electrodes with different pre-treatments, **b**: charged electrodes in ‘a’ discharged with 0.1 mA constant current, **c**: charged electrodes in ‘a’ discharged with 0.5 mA constant current, **d**: charged electrodes in ‘a’ discharged with 1.0 mA constant current).

Once applied in MBs, all pre-treated solid-state electrodes acted as electron sink for power output (Fig. 4a), which was the direct proof that CB can be an alternative material in MBs. Moreover, the varied current curves demonstrated that different electrochemical capacities of CB were obtained through different treatments. Further quantitative analysis (Table 1, average values calculated from Fig. 4 and Fig. S8) revealed that unsintered CB exhibited the highest electron equivalent capacity of 18.58 ± 0.46 C/g. Thermal treatment was prejudicial to the preservation of capacity. CB sintered with N_2_ displayed the poorest capacity of 2.29 ± 0.48 C/g, while sintering with O_2_ was relatively conducive to maintaining electron-acceptance, with a capacity of 8.97 ± 0.19 C/g obtained under 70% O_2_ and 30% N_2_ and 8.26 ± 0.13 C/g under 30% O_2_ and 70% N_2_. XPS results showed that the contents of quinone C=O moiety obtained by different treatments on CB corresponded to their different capacities, i.e., the unsintered electrode contained the highest percentage of 63.2%, while the percentage significantly decreased to 27.6%, 22.8%, and 10.8% after thermal treatment as the proportion of N_2_ in the sintering atmosphere was increased (Fig. 3a–d). The correlation between CB’s capacity and its C=O moiety abundance proved the role of quinone C=O as electron sink that was indicated by previous LSV characterization, and also proved the indication in our study of a moiety-based mechanism for CB’s feasibility as a cathode material in MBs.

**Table 1:** Equivalent electron capacity of BC-based electrodes with different pre-treatments (‘0.1mA-discharged’, ‘0.5mA-discharged’, ‘1mA-discharged’ represent charged electrodes discharged with 0.1, 0.5 and 1.0 mA constant current, respectively).

	Capacitance (C/g)^a^			
	newly-fabricated	0.1mA-discharged	0.5mA-discharged	1.0mA-discharged
100%N_2_ sintered	2.29 ± 0.48	4.75 ± 0.16	5.99 ± 0.18	7.02 ± 0.86
O_2_:N_2_ = 3:7 sintered	8.26 ± 0.13	9.29 ± 0.10	9.40 ± 0.05	9.70 ± 0.03
O_2_:N_2_ = 7:3 sintered	8.97 ± 0.19	9.36 ± 0.051	9.45 ± 0.127	10.93 ± 0.644
unsintered	18.58 ± 0.46	19.33 ± 0.95	19.54 ± 1.00	20.42 ± 2.01

^a^Shown are average values calculated from measurements of replicate BC-based electrodes (shown in Fig. 4 and Fig. S10) ± standard deviations.

However, as a material aimed to be practically used in MBs for charge storage, we found the specific capacity of CB in our work is far from satisfying the practical application from weight and size perspectives, especially compared to metal materials normally used in the cathode of conventional batteries (Table 2)^33, 34^. Compared with the conventional battery completely focusing on the energy storage, MBs aim at energy acquirement as well as wastewater treatment as organic reservoirs. Thus the superior properties in terms of economy, biocompatibility and anti-corrosion make carbon materials the better candidates applied in MBs if their specific capacities can be improved. Meanwhile, we learnt the capacity storage of CB was determined by various factors such as surface structure, surface area, surface functionalities and some of them or some other carbon materials have even shown the specific capacity comparable to normally used metal materials (Table 2)^33–37^. Thus efforts including changing the configuration of electrode to increase its surface area, doping of active element (such as O, S) to further improve active sites of CB may potentially enhance the specific capacity of CB to truly satisfy its application in MBs. And these are what we further concentrate on to expand our current research to an application area.

**Table 2:** Specific capacities of typical materials.

	Material type	Specific capacity (c/g)	Reference
Carbon-based material	Carbon black (C-MA21)	7.7 × 10^−3^	37
	Carbon black (C-LIMT)	0.32	37
	Carbon black (C-SMT)	144.0	37
	Carbon black (VXC 72 R)	18.6	This study
	Graphene networks	246.0	36
	Carbon nanotubes	720.0	37
Metal material	Li/Li^+^ (a)	−0.9	33
	NiO/Ni(OH)_2_ (b)	2880	34

(a) Most widely studied and applied battery material.

(b) Material with the best-published specific capacity result in the literature.

### Regeneration of CB Cathodes

The recyclable utilization of cathode is another element for the practical application, which means CB-based electrodes should be regenerated once subjected to electron saturation. As expected, CB’s electron-capturing ability could be reproduced by discharging the CB samples through a reverse electrochemical oxidation (Fig. 4b–d). The re-charging current curves of the discharged CB samples significantly varied depending on the discharge parameters, i.e., 0.1-mA (Fig. 4b), 0.5-mA (Fig. 4c), or 1.0-mA constant current (Fig. 4d). The greatest influence was observed on the CB electrode sintered in N_2_ only, the capacity of which doubled after it was discharged at 0.1 mA constant current and further grew to 5.99 ± 0.18 C/g after being discharged at 0.5 mA and 7.02 ± 0.86 C/g at 1.0 mA. Despite the constant increase in capacity induced by the increased discharge current, the highest growths (at 1.0 mA discharging current) of only 9.9% for the unsintered, 21.8% for 30% O_2_ sintered, and 16.81% for 70% O_2_ sintered CB was obtained, respectively. Given the relatively low amount of C=O on the initial 100% N_2_ sintered CB, the sharp capacity increase was supposed to have derived from electrochemical activation of functional moieties acting as the electron sink, while the other treated CBs possessed relatively more active moieties, which were not prone to activation. Indeed, the XPS results showed that the C=O functional moieties of different electrode categories all recovered, to 59.6% for the unsintered CB, 30.6% for the 100% N_2_ sintered CB, 50.5% for the 30% O_2_ sintered CB, and 53.3% for the 70% O_2_ sintered CB (Fig. S9). The electrode sintered with pure N_2_ exhibited the largest increase in C=O moiety abundance by discharging, which was the direct proof of its maximum capacity increase after electrochemical oxidation. The above results prove CB for MB application can be reversed by electrochemical reoxidation through C=O moiety recovery or potential activation and CB’s capacity recovery is decided by electrochemical parameters.

**Figure 5:**
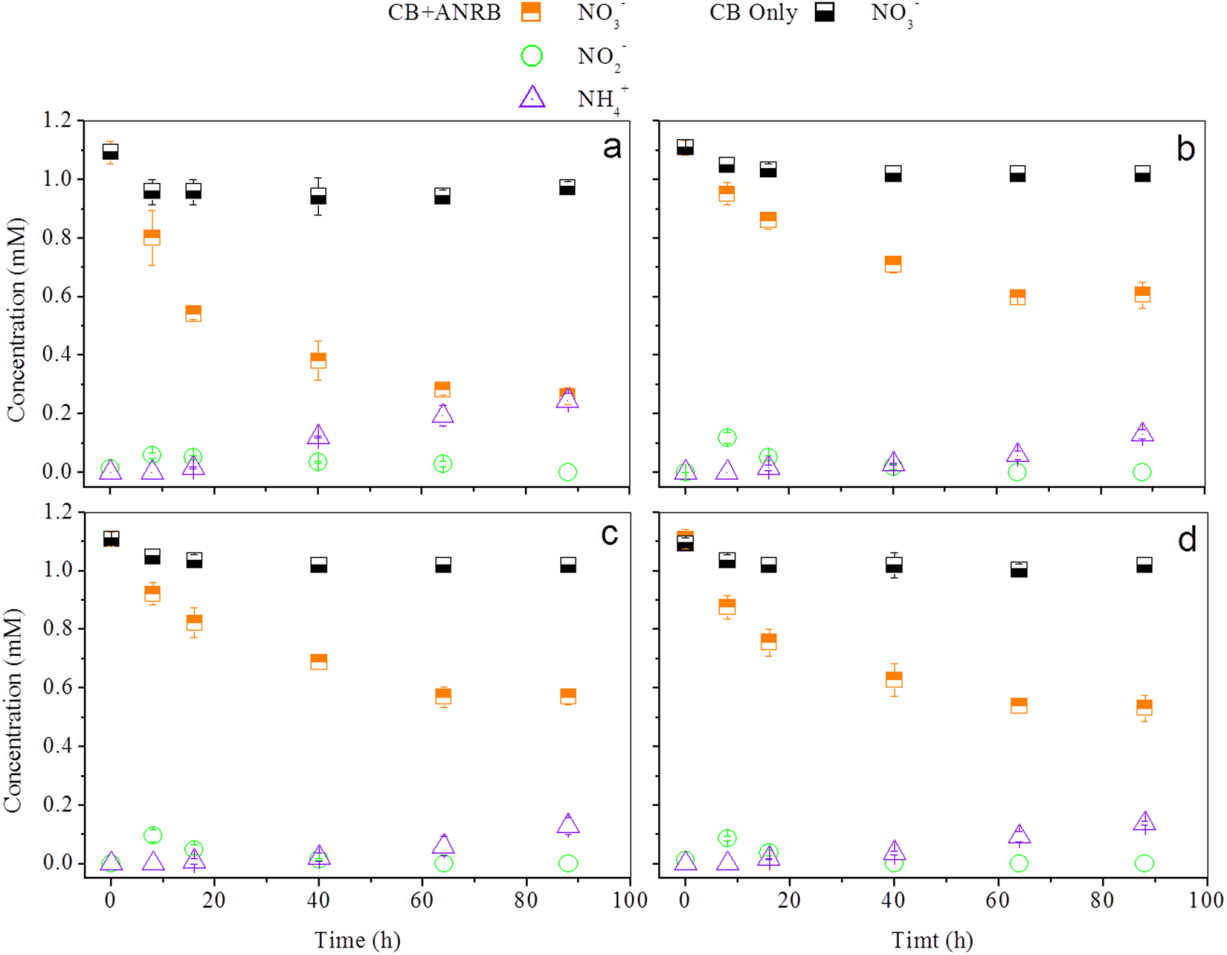
Nitrate reduction with charged CB electrode as electron donor. (**a**) electrodes unsintered were applied, (**b**) electrodes sintered under 100%N_2_ were applied (**c**) electrodes sintered under 30%O_2_ and 70%N_2_ were applied, (**d**) electrodes sintered under 70%O_2_ and 30%N_2_ were applied (ANRB: autotrophic nitrate-reduction bacteria, each data was reported as average value from replicate CB electrodes and triple measurements).

Compared to direct electrochemical regeneration of CB, another desirable strategy is exposing CB to some oxidant. Here in our study nitrate was chose as the potential activator for CB’s electron-accepting capability. As shown in Fig. 5, nitrate was slightly removed (removal efficiency ranging between 6.8% and 11.0%) within the first 8 h, without nitrite and ammonia accumulation throughout the whole reaction process of 88 h when no autotrophic nitrate-reduction bacteria (ANRB) were inoculated in the CB control systems, indicating limited sorption of nitrate by bare CB electrode in this study. In ANBR-control, nitrate was removed instantly but only to a limited extent (23.6%), with slight nitrite and ammonium accumulation detected (Fig. S11). This indicated that some nitrate could be reduced by ANBR deriving electrons from itself, the phenomenon of which could be clarified by the knowledge that many species store electrons in their periplasmic and outer-surface cytochromes^38, 39^. As ANRB were combined with charged CB, nitrate was constantly removed over 88 h with impressive accumulation of ammonia and gradual depletion of nitrite after its initial peak during the first 8 h of the experiment (Fig. 5a–d). These results suggested that electron-saturated solid-state CB electrodes can serve as electron donors for microbial nitrate reduction and microbial-catalyzed nitrate activation can meet the potential criterion for the recovery of CB’s electron-capturing ability.

Moreover, nitrate experienced different reduction kinetics when CBs with different pre-treatments were used as electron donors. It indicated that the nitrate removal rate and amount depended on the capacities of CBs, i.e., unsintered > sintered under 70% O_2_ and 30% N_2_ > sintered under 30% O_2_ and 70% N_2_ > sintered under 100% N_2_ (Fig. 5). Unsintered CB exhibited the fastest nitrate removal rate and the highest removal efficiency of 76.2% in 88 h (Fig. 5a), while the 100% N_2_ sintered CB exhibited the slowest removal rate and the lowest removal efficiency of 45.4% (Fig. 5d).

The ability of CB to act as an electron sink could be recovered by donating electrons to nitrate (Fig. S12). After nitrate reduction, CB electrodes were reinstalled to MBs for measurements. The current curves based on different CBs, regardless of pre-treatments, closely matched those of their initial charging curves (Fig. S12), and their capacities were almost recovered (Table S4). Meanwhile, an evaluation of capacity balance was conducted to reveal the fate of electrons donated by CBs (the specific calculation of capacity balance can be found in Supplementary Information). According to the observed ammonium formation and nitrate loss, around 120.0% capacity was calculated to have participated in nitrate reduction for all CB electrodes (Table S4). This slight exceeding of the 100% capacity contribution of CB to nitrate reduction was probably attributable to nitrate’s potential reduction flux into other products such as nitrogen monoxide or nitric oxide, both of which experience less electron transfer per unit nitrogen reduction. Although this topic deserves further study, these figures strongly hint that the solid-state CB electrodes can be fully regenerated by donating electrons to nitrate reduction through a favorable microbial catalysis process.

Compared with the regeneration method of electrochemical oxidation, the strategy based on transferring electrons to oxidant seems to be more favorable, as no extra electrical energy would be consumed by this spontaneous redox reaction. Moreover, compared to oxygen being regarded as regeneration activator, feasibility of recovery by nitrate reduction yields more significant applied engineering implications. By a spontaneous microbial respiration process, ideally, electrons can be harvested from wastewater^2, 40^ in MBs and stored in CBs, through which bioavailable electrons become mobile. In the long run, these mobile ‘electron storerooms’ can then be applied to wider *ex-situ* remediation of pollutants (such as the reduction of heavy metal Cr(VI)^41^, dechlorination of chloride compounds^42^) in sediments or wastewater plants. Moreover, as this step happens outside MBs, parameters like temperature, pressure, and electrolyte composition can be optimized for practical application.

### Resilience and Persistence of Chemical Activity of CB Electrode

**Figure 6:**
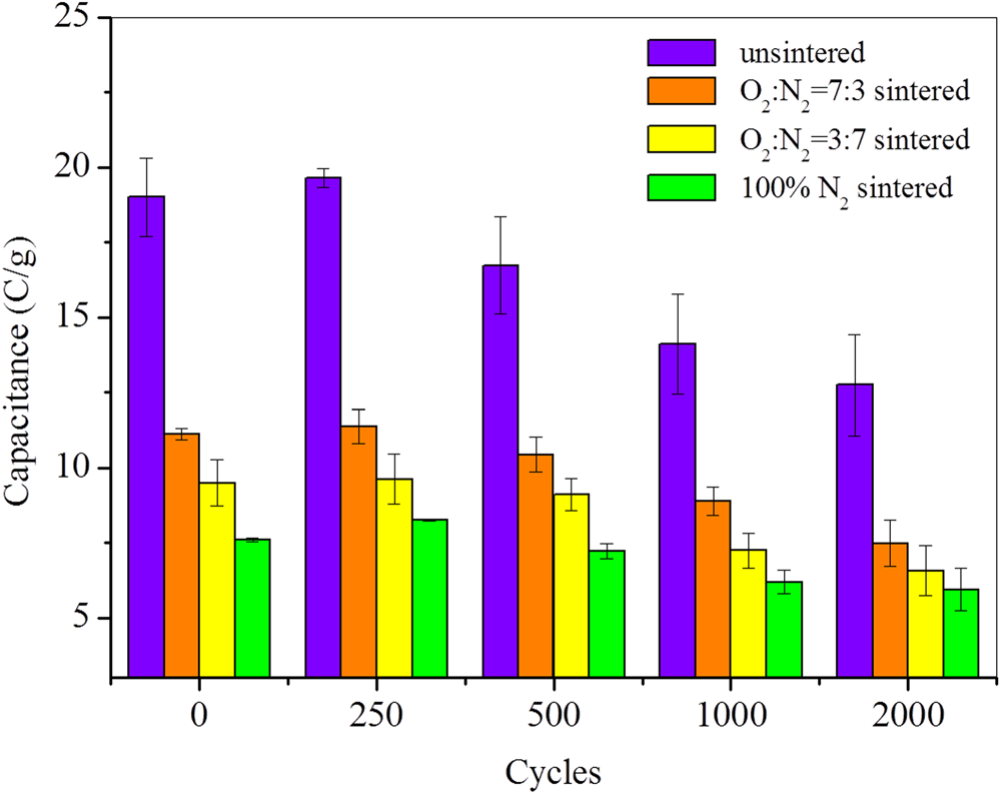
Equivalent electron capacities of CB electrodes with different charging-discharging cycling.

Lifetime in terms of resilience and persistence is another important concern for the cathode of MB for its practical application. Current curves showed that the electron accepting capability of CB could be reversed after reduplicated charge–discharge cycles (Fig. S13), indicating a good resilience and persistence of its chemical activity. Further calculations revealed that the capacities of all CB electrodes were fully preserved during the initial 250 cycles (Fig. 6). CB electrodes experienced a constant decrease in capacity after 2000 cycles, with reductions of 32.9%, 32.7%, 30.8%, and 21.8% for unsintered CB, CB sintered under 70% O_2_ and 30% N_2_, CB sintered under 30% O_2_ and 70% N_2_, and CB sintered under 100% N_2_, respectively (Fig. 6). Given the potential activation of capacity by electrochemical processes indicated by the results above, this fall in capacity was probably caused by the annihilation of some active surface moieties. Considering the easy accessibility as well as low-price of carbon materials, these capacity loss is relatively acceptable from a practical application consideration.

## Conclusions

The results of this work confirm the chemical activity of CB and prove its potential as a promising alternative by its low-cost, easy-regeneration and resilience for the solid-state cathode in MBs. CB is potential-favorable to derive electrons from biodegradable organics by microbial extraction. The coincidence between the capacity of CB cathode and its abundance of C=O moiety indicates CB’s role as electron sink derived from redox-active C=O functionality. Reduced BC in the cathode of MBs can be recovered by electrochemical reoxidation through a reversible C=O moiety shift. Besides, this study suggests CB cathode of MBs can also be regenerated by a more desirable method through *ex-situ* microbial-catalyzed redox reaction to release electrons to nitrate.

## Methods

### Chemicals and Materials

A finely powdered highly conductive carbon black, Cabot VXC 72 R CB (surface area: 254 m^2^/g, average primary particle size: 30 nm, DBP-absorption: 192 cc/100 g, density: 96.1 g/L) was purchased from Cabot China Inc. and used as received. PTFE suspension (60 wt%) was received from Hesen Electrical Co., Ltd, Shanghai, China. All other chemicals were of analytical reagent grade and obtained from Changqing Chemical Co., Ltd, Hangzhou, China.

### Preparation of CB electrodes

The preparation of the CB electrodes was conducted according to a method modified from a previous report^12^ (briefly described in Fig. S14): First, 40 wt% of CB was dispersed into 20 times the weight of ethanol in a beaker and ultrasonically agitated for 30 min at 30 °C. Next, 60 wt% PTFE suspension was dripped into the blend slowly and the mixture was ultrasonicated for another 30 min to fully disperse the carbon black and PTFE particles. The blend was then stirred and dried in an 80 °C bath to produce a dough-like paste. The paste was then molded and rolled into an even sheet electrode (circular in shape with a diameter of 3 cm and thickness of 2 mm). Afterwards, electrodes were treated with different methods including naturally dried at room temperature or sintered at 340 °C for 30 min under different atmospheres: 100% N_2_, N_2_ and O_2_ mixed at the volume ration of 3:7 and 7:3 respectively. The sintering was carried out in a sealed tubular furnace (GWL-1400xa, Juxing Ltd., Luoyang, China) under a constant gas flow rate of 400 mL/min. The purpose of these diverse treatments is to form CB with different moiety characters to identify the relation between functionality and electron-capturing capability.

### Redox Characterizations of CB electrodes

Linear sweep voltammetry (LSV) and cyclic voltammetry (CV) were used to investigate the electrochemical redox properties of CB electrodes with the electrochemical workstation (Biologic VSP, Claix, France). Both measurements were conducted in a three-electrode configuration, with a newly manufactured or totally charged CB electrode as the working electrode, a platinum electrode as the auxiliary electrode, and an Ag/AgCl reference electrode. For LSVs, the potential was scanned from the open circuit potential to −1.0 V (vs Ag/AgCl electrode) at a scan rate of 1.0 mV s^−1^. CVs were conducted at the potential window from −1.5 V to 1.5 V and at the scan rate of 1.0 mV s^−1^.

### Electrochemical measurements in MBs

The configuration of MB was as described previously^43^. The detailed configuration and schematic diagram of the MB were presented in the Supplementary Information (Fig. S2). Anode electrodes were primarily pre-colonized for 15 d in a high-throughput bioelectrochemical system (BES) reactor^44^, in which several anodes shared the same cathode and the reference electrode. A mixture of modified M9 medium (NH_4_Cl, 0.1 g/L, NaCl, 0.5 g/L, KH_2_PO_4_, 4.4 g/L, K_2_HPO_4_, 3.4 g/L, MgSO_4_, 0.1 g/L, NaHCO_3_, 2 g/L) with trace elements and 1.5 g/L sodium acetate was used as the anodic medium^45^. Meanwhile, an exoelectrogens, i.e., *Geobacter*, pre-enriched in our laboratory^43^, was inoculated as the inoculum. M9 medium with 10 g/L potassium ferricyanide was used as the cathodic electrolyte. Once the anodes had reached their stable current intensities, they were reinstalled to the MBs described above.

For measurements, M9 with 1.5 g/L sodium acetate was used as the anodic electrolyte, while M9 solution without potassium ferricyanide was used as the cathode electrolyte, with the fabricated CB cathodes as potentially the sole electron acceptors. MBs were operated under anaerobic conditions without the presence of oxygen as potential electron acceptor. An external resistance (1 ohm) was applied to connect the anode and cathode for current collection using a data acquisition system (34970 A, Agilent, America). The electron capacity of the CB samples was calculated by integrating current profile according to the formula: *C* = ${\int }_{0}^{T}{Itdt}$, where I is the current collected and T is the time when MBs were approaching current values of zero.

### Regeneration of CB electrodes by electrochemical oxidation

Once the CB electrodes were totally “charged” (fully saturated with electrons from the organics and approaching no current output) in MBs, their regenerations were tested by discharging in a three-electrode configuration^46^, with platinum auxiliary electrode and Ag/AgCl reference electrode using an electrochemical workstation mentioned above. The discharging process was conducted in modified M9 solution for 1 h at a constant current of 0.1 mA, 0.5 mA, and 1.0 mA, separately. Then discharged CB electrodes were re-migrated to MBs to evaluate their electron-capture abilities.

### Regeneration of CB electrodes by exposure to nitrate

Once the CB electrodes had been fully “charged” in MBs, they were anaerobically transferred to a 100 mL anaerobic culture bottle with a working volume of 50 mL. M9 without NH_4_Cl was used as the medium, and 1.1 mM NaNO_3_ was added as the potential electron acceptor. The medium was inoculated with pre-enriched autotrophic nitrate-reduction sludge (specific enrichment method and functional effectiveness seen in Supplementary Information) at a mixed liquid sludge solids concentration of 300 mg/L for autotrophic nitrate reduction with the charged BC electrode as the sole electron donor. The inoculated medium was first aerated in a mixture of N_2_ and CO_2_ gas (v/v = 80/20) for 30 min and then cultured anaerobically at 30 °C in oven oscillator (SKY-2102C, Shukun, Ltd., Shanghai, China) operated at 160 rpm. As soon as the reduction reaction ceased, the discharged electrodes were reinstalled to MBs to study the recovery of their electron-capture property.

### Resilience and persistence tests

The resilience and persistence of the chemical activity of the CB electrodes were tested by evaluating their inherent electron capacities after repeated charging–discharging processes, to show if CB electrodes are robust for long-time operation. The charging-discharging cycles were performed using the three-electrode configuration as for the reversibility tests. The galvanostatic cycling with potential limitation (GCPL) module of the electrochemical workstation (Biologic VSP, Claix, France) was used to achieve 250, 500, 1000, and 2000 charge–discharge cycles, each with a stable potential as terminal discharging and charging platform. At the end of the cycling process, the CB electrodes were reinstalled to MBs and then tested.

### Fourier transform infrared spectroscopy (FTIR)

FTIR analysis was performed on a Fourier transform infrared spectrometer (Vertex 70, Bruker, Germany) in the frequency range 4500–500 cm^−1^.

### X-ray Photoelectron Spectroscopy (XPS)

XPS spectra were collected using an EscaLab 250Xi spectrometer with a monochromated Al Kα source (Thermo, UK). Spectra calibrated on the 538.1 (O1s) and 296.1 (C1s) eV peaks were analyzed using XPSPEAK (version 4.1) software.

## Electronic supplementary material

Supporting Information
