# Supplementary material for: Carbon black as an alternative cathode material for electrical energy recovery and transfer in a microbial battery
Source: Sci Rep. 2017 Aug 1;7:6981. doi: 10.1038/s41598-017-07174-z (PMC5539158; doi:10.1038/s41598-017-07174-z)
Supplement: Supplementary file 1 — Supporting Information [file 41598_2017_7174_MOESM1_ESM.pdf]

Carbon black as an alternative cathode material for electrical energy recovery and transfer in a microbial battery

Xueqin Zhang[1,2,a], Kun Guo[3,a], Dongsheng Shen[1], Huajun Feng[1]*, Meizhen Wang[1]*, Yuyang Zhou[1], Yufeng Jia[1], Yuxiang Liang[1], Mengjiao Zhou[1]

[1]Zhejiang Provincial Key Laboratory of Solid Waste Treatment and Recycling, School of Environmental Science and Engineering, Zhejiang Gongshang University, Hangzhou, 310012, China; [2]Advanced Water Management Centre, The University of Queensland, St Lucia, QLD 4072, Australia; [3]Center for Microbial Ecology and Technology, Ghent University, Coupure Links 653, B-9000, Ghent, Belgium

Correspondence and requests for materials should be addressed to Fenghuajun@mail.zjgsu.edu.cn or wmz@zjgsu.edu.cn.

Electrochemical test of CB's redox characterization for hydrogen evolution

Newly fabricated CB electrodes were firstly relocated to the cathode of reactors with configuration the same to MB reactors for electrochemical measurement in our study (Fig. S1). M9 was used as anodic and cathodic electrolyte. The working volume of cathode chamber was dominated to be 40 mL, with another 10 mL to be the headspace volume. A constant cathode potential of -1.6 V (vs Ag/AgCl) was applied using an electrochemical workstation (Biologic VSP, Claix, France) for sustainable electrode charging process (seen in Fig. S2). The gas composition prior (initial status) and after charging (as soon as current was decreased to be negligible) was determined using a gas chromatograph (GC7890-II, Tianmei Co., Shanghai, China).

As shown in Fig. S2, currents for electrodes with different pre-treatments all saw constant drop, indicating a diminished electron capability in the cathode chamber. It implied that electrons in the cathode were not likely accepted for hydrogen evolution as electrons would be sustainably utilized in the cathode for this fate then current could be constantly maintained at a considerable level.

Moreover, we used calculation results to further figure out where electrons were finally directed to. Based on Fig. S2, total electrons amount accepted in the cathode can be calculated according to formula (details can be found in Table S1):

$$E = \frac{\int_0^T Itdt}{F}$$

where *I* was the current collected, *T* was the time when current was negligible, *F* was the Faraday Constant (96487 C/mol)

As no other liquid electron acceptor was added in the cathode chamber, we assumed that all electrons transfer observed derived from hydrogen evolution. Based on reaction equation for hydrogen evolution,

$2H^{+} + 2e^{-} = H_2$

hypothetic hydrogen production was calculated to be $0.5E$ (the separated production for differently treated electrodes can be found in Table S1).

In the sealed cathode chamber, whole hydrogen produced was dispersed to the headspace or held in the liquid phase by dissolution. Thus hydrogen amount partly accumulated in the headspace could be calculated according to the Dicipline of Henry:

$P_{(H2)} = H_{(H2)} \times C_{(H2)}$

where $P_{(H2)}$ was the partial pressure of hydrogen in the headspace, $H_{(H2)}$ was the Henry coefficients for hydrogen in the water solution at the temperature of 30°C (7.39×10^{5} KPa), $C_{(H2)}$ was the mole ration of hydrogen in the solution.

Based on total hydrogen production, hydrogen accumulated in the headspace (including amount and volume fraction) was calculated and the detail results for differently treated electrodes could be found in Table S1. Obviously, based on assumption, considerably measurable hydrogen (ranging between 4.8% and 29.3%) would be detected in the headspace, while actually no hydrogen was traced for all pre-treated CB electrodes working as redox mediates. These results confirmed that CB itself can act as electron sink prior reduction reaction for hydrogen evolution.

However, when CB-based electrodes were charged, reduction tendencies

re-appeared while the potential located at region lower than -1.8V (Fig. S3). As no reductive peaks were observed at potential more positive than -0.8 V for charged electrodes (Fig. 1b), the reduction tendencies located at region more negative than -1.8V seemed to derive from hydrogen evolution. This speculation was confirmed by the observation of bubble on the surface of charged CB electrodes when CV experiments were conducted and the traceable hydrogen detection in the headspace when charged CB electrodes were oriented at -2.0V. These results further proved CB's activity by no observation of hydrogen evolutions on the surface of uncharged electrodes.

Detailed Analysis of Fourier Transform Infrared Spectroscopy (FTIR)

The FTIR profiles of CB powder and CB-based electrodes are shown in Fig. S5 and Fig. 2, respectively, and the corresponding band assignments are listed in Table S2. Initial CB powder showing the absorbance of the dominant O-H stretch at 3200-3500 cm^{-1} hinted the adsorption of water molecules by CB[7,8]. Meanwhile it showed notable vibration of C=C at 1440 cm^{-1} indicative of the existence of aromatic C[1,2] and vibration of C=O at 1630 cm^{-1} indicative of the strong presence of quinone structures[2,3], both of which indicated the CB as an excellent electron sink. This is the direct proof of CB's chemical activity.

Noteworthy bands shifts were observed as CB is fabricated into electrodes. The most predominant vibrations at approximately 1200 cm^{-1} belong to C-F stretching[4], which was due to the introduction of the binder of PTFE for the electrode fabrication. Similarly, the new vibration at around 2825-3000 cm^{-1} featuring C-H stretching was also attributed to the introduction of PTFE[7]. Furthermore, compared to CB, O-H stretch was shifted to band around 3500-3800 cm^{-1}, which is regarded as a 'free' O-H pattern in terms of alcoholic –OH but not hydrogen bonded[7,8]. This was probably resulted from the introduction of ethanol during the electrode fabrication procedure. In addition, unsintered CB-based electrode totally preserved the CB's absorbance featured unsaturated C=C band at 1440 cm^{-1} and C=O at 1630 cm^{-1}, confirming its capability of electron-accepting as a cathode in MBs.

Referring to sintered electrodes, the three pre-treated under different conditions showed the same absorbance with the differences on the vibration strength. Compared

with unsintered electrode, C-F and C-H stretching aroused by PTFE binder was maintained as expected; ‘free’ O-H at 3500-3800 cm^{-1} was preserved but loose intensity which was likely due to absorbed-ethanol volatilization by thermal treatment. Although quinone C=O band was preserved, unsaturated aromatic C=C (at 1440) disappeared after sintering, indicating the partial reduction of CB by thermal treatment[14]. This explained the weakened capacity of sintered CB electrode.

To summarize, FT-IR spectra showed (i) CB’s chemical activity likely derives from the surface functionalities including unsaturated aromatic C=C and quinone C=O, which acted as electron sink, (ii) unsintered CB-based electrode wholly preserved CB’s absorbance featured unsaturated C=C band and C=O while only quinone C=O band was remained for differently sintered electrodes.

Detailed Analysis of X-ray Photoelectron Spectroscopy (XPS)

Here in our samples oxygen-containing functionalities were the source of CB's chemical activity, thus quantitative characterizations of the remaining oxygen-containing functionalities were performed using XPS analysis (As the high-proportion C element was blended into fabricated electrodes by the introduction of PTFE and the blending amount was unavoidably uneven for different electrodes, we found the relative abundance of different carbon-based functionalities in different electrodes significantly varied, thus the same carbon-based functionality in different electrodes was not comparable. So the results of C1s resolution were not presented and analyzed here). Deconvolution of O1 curve for CB powder resulted in two noteworthy peaks centered at 532.1 and 533.5 eV and they could be attributed to the C-O and C=O group, respectively (Fig. S6 and Table S3)[9,10]. Unsintered CB electrode preserved the same functionalities of C-O and C=O, even though the binding corresponding to C=O was shifted to 531.1 eV (Fig. 3 and Table S3)[10]. The O1s of sintered CB electrodes, regardless of atmospheric conditions, could be fitted into three peaks at 531.3, 532.3 and 533.0 eV, with each assigned to C=O, C-O and C-OH group, separately (Fig. 3 and Table S3)[9,10]. Previous studies have proved that functionalities on the surface of carbon materials including hydroxyl, carbonyl, carboxyl, and lactone groups can lead to reversible redox reactions, which can be expressed as follows[14,15]:

$$> \text{C-OH} \rightleftharpoons > \text{C} = \text{O} + \text{H}^+ + \text{e}^- \quad (1)$$

$$-\text{COOH} \rightleftharpoons -\text{COO} + \text{H}^+ + \text{e}^- \quad (2)$$

$$> \text{C=O} + \text{e}^- \rightleftharpoons > \text{C-O}^- \quad (3)$$

Thus, the C=O group was an excellent electron sink and it proves the chemical activity of CB as well as the capability of CB-based cathode for electron-accepting in MBs.

Further quantitative characterizations revealed that the percentage of C=O for unsintered electrode is 63.2%, while it significantly decreased by thermal treatment for sintered electrodes, with its percentage of 10.8% for electrode sintered under 100% N_2, 22.8% for electrode sintered under 30% O_2 and 70% N_2 mixed atmospheric condition and 27.6% sintered under 70% O_2 and 30% N_2 mixed atmospheric condition (Fig. 3). It was noteworthy that C=O percentage decreased with the oxygen proportion drop in the sintering gas, while the C-OH group at 533.0 eV increase accordingly. These results further proved the thermal treatment results in partial reduction of CB by unsaturated C=O transformed into saturated C-O and this process was favorably catalyzed by a reductive reaction environment.

Previous results[16,17] indicated the source of electron sink for CB-based electrodes likely come from the C=O group at 531.3 eV, and this was further confirmed by the percentage decline of C=O group while electrodes were totally charged in MBs (Fig. S7). Thus the appearance of group C-OH at binding 533.6 was anticipated to be the new product of electron trap by sintered electrodes. Interestingly, the ration diversity of oxygen-containing functionalities for electrodes sintered under different conditions was completely eliminated by ‘charging’ process, leading to the similar component (including group and percentage) of oxygen-containing functionalities. This meant electrodes sintered under different conditions were all saturated with electrons. For

unsintered electrode, charging also processed with C=O percentage drop at 531.3 eV, resulting in dismutation generating new groups of C-O at 529.9 eV, C=O at 530.7 eV and C-O at 531.7 eV.

Equation (1)-(3) also clarify the potential pattern of CB's reversibility and we simultaneously applied the XPSs of discharged (with 1.0 mA constant current) samples to elucidate the reversible mechanism. As shown in Fig. S9, the C=O group at 531.3 eV (or 531.1 eV), was evidently recovered, the percentage of which provided 59.6% for unsintered CB-based electrode, 30.6% for electrode usintered under 100% N_2, 50.5% for electrode sintered under 30% O_2 and 70% N_2 mixed atmospheric condition and 53.3% sintered under 70% O_2 and 30% N_2 mixed atmospheric condition. As was the electron sink here, C=O group recovering by discharging provided the direct proof of the reversible property of CB as cathode material for MBs. Also the extent of the C=O group recovery confirmed the dramatic capacity increase for electrodes sintered under 100% N_2 (which was speculated to be the activation of some functional groups), but gentle growth for electrodes with the other treatment since electrodes are discharged by 1.0 mA constant current (Table 1).

Altogether, XPS results showed: (i) CB-based cathode acted as electron sink in MBs by the surface functionality of C=O, (ii) CB's chemical activity in terms of capacity was determined by pre-treatments shifting active C=O group into different percentage, (iii) the reversible property of CB depended on the recoverable nature of C=O group.

Acclimation and Enrichment of Autotrophic Nitrate-reduction Bacteria

Autotrophic nitrate-reduction cells were acclimated and pre-enriched by providing nitrate as the sole nitrogen source and hydrogen as the sole electron donor (as well as energy source). Sludge collected from the settling section of Qige Domestic Sewage Treatment Plant (Hangzhou, China) was inoculated as inoculum. M9 replacing NH_4Cl with $NaNO_3$ was used as the medium. After washing with medium for triple, sludge was relocated into a 500 mL BPN Culture Bottle, with the mixed liquid sludge solids concentration of 3000 mg/L, and cultured anaerobically at 30℃ in a 160 rpm working oven oscillator (SKY-2102C, Shsukun, Ltd., Shanghai, China). The headspace (about half of the bottle volume) was replace by H_2 and CO_2 mixed-gas (v:v=80:20) prior to culture in oven oscillator and refreshed every 24 hours. $NaNO_3$ was also added every 24 hours with increasing concentration gradient from 0.5 mmol/L to 10 mmol/L in 30 days and the medium was refreshed every 4 days to avoid alkalization of solution due to OH^- accumulation by nitrate reduction reaction. The nitrate removal kinetics on day 30 can be seen in Fig. S10.

Obviously, nitrate could be efficiently removed within 18 h, with significant total nitrogen decrease and partial ammonia nitrogen accumulation. It demonstrated the successful enrichment of autotrophic nitrate-reduction bacteria in our experiment.

Calculation of CB's Capacity Balance for Nitrate Reduction

Here we supposed nitrogen and ammonium were fully two final production of nitrate reduction. According to Fig. 5 and Fig. S11

, based on nitrate losses and ammonium formation,

a) Capacity corresponding to electrons for ammonium release

   = (NH_4^+ formed in CB+ NRB reactors − NH_4^+ formed in NRB-only control) × (solution volume (100 mL)) × (number of e^- transformed per nitrate (8)) × (Faraday constant (96487 C/mol))

b) Capacity corresponding to electrons for nitrogen release (assuming other nitrate losses was transferred to N_2)

   = (nitrate loss in CB+ NRB reactor reactors − nitrate loss in NRB-only control − nitrate loss in CB-only control −（NH_4^+ formed in CB+ NRB reactors − NH_4^+ formed in NRB-only control)）× (solution volume (100 mL)) × (number of e^- transformed per nitrate (5)) × (Faraday constant (96487 C/mol))

The capacity balance is calculated by estimating CB's capacity contribution rate (CCR) for nitrate reduction through the following formula:

CCR = (Capacity (a) + Capacity (b)) ÷ Capacity of CB-based electrodes possessing

The detailed results of capacity balance are shown in Table S4.

Table S1. Evaluation of CB's redox characterization for hydrogen evolution

	100% N_2 sintered	O_2:N_2=3:7 sintered	O_2:N_2=7:3 sintered	unsintered
Capacity equivalent for electrons transfer observed (C)	3.49	11.61	12.29	21.21
Expected total hydrogen amount evolved (mL)[a]	0.81	2.70	2.85	4.93
Expected hydrogen amount released in headspace (mL)[b]	0.48	1.61	1.70	2.93
Expected hydrogen volume fraction in headspace (%)	4.8	16.1	17.0	29.3

[a] Theoretical total hydrogen evolution calculated from whole electron transfer (Fig. S2); [b] Theoretical hydrogen evolution partially released to headspace based on Henry Law.

Table S2. Assignment of characteristic vibrations to individual peaks for CB powder and CB-based electrodes pre-treated under different conditions

Samples	Wavenumber (cm^{-1})	Characteristic vibrations	Functionality
CB	1440	C=C	aromatic C, appears when bound to unsaturated group[1,2]
	1630	C=O	Quinone[2,3]
	3200-3500	O-H	water, H-bonded hydroxyl (-OH) groups[1,2]
Unsintered	1200	C-F	C-F stretching[4]
	1375	O-H	phenolic -OH, related to ligneous syringyl units[5]
	1440	C=C	aromatic C, indicative of lignin, appears when bound to unsaturated group[1,2]
	1630	C=O	Quinone[2,3]
	1710	C=O	Mainly carboxyl; traces of aldehydes, ketones and esters[3,6]
	2825-3000	C-H	Aliphatics[7]
	3500-3800	-OH	alcoholic -OH, not hydrogen bonded[7,8]
Sintered (100%N_2, N_2:O_2=3:7, N_2:O_2=7:3)	1200	C-F	C-F stretching[4]
	1375	O-H	phenolic -OH, related to ligneous syringyl units[5]
	1550-1630	C=O	Quinone[2,4]
	2850-3000	C-H	Aliphatics[6]
	3500-4000	-OH	alcoholic -OH, not hydrogen bonded bonded[7,8]

Table S3. Peak assignments for O forms obtained from O (1s) XPS

	Samples	Binding energy (eV)	Elementary Form	Functionality
Initial	CB	532.1	C-O	hydroxyls, ethers[9]
		533.5	C=O	carbonyl, quinone[10]
	Unsintered	531.1	C=O	esters, anhydrides quinone, carbonyl[10,11]
		532.1	C-O	hydroxyl, ethers (Valdes et al., 2002)
	Sintered Electrode	531.3	C=O	quinone[10,11]
		532.3	C-O-C	ether, hydroxyl[10]
		533.0	C-O/C-O-C	ether[12]
Charged	Unsintered	529.9	C-O	hydroxyls, ethers[9]
		530.7	C=O	carbonyl, quinone[9]
		531.1	C=O	Quinone[10,11]
		531.7	C-OH	Carbonyl[12]
		532.1	C-O-C/C-OH	ether, hydroxyl[9]
		533.0	C-O/C-O-C	ether [12]
	Sintered	531.3	C=O	Quinone[10,11]
		532.1	C-O	ether, hydroxyl[9]
		533.6	C-OH/C-O-C	esters, anhydrides[13]
Discharged	Unsintered	531.3	C=O	Quinone[10,11]
		532.7	C-O/C-O-C	Carbonyl[12]
		533.6	C-OH/C-O-C	esters, anhydrides [13]
	100%N_2 sintered	531.1	C=O	Quinone[10,11]
		532.3	C-O-C	ether, hydroxyl[10]
		532.9	C-O/C-O-C	ether, hydroxyl[12]
	Sintered (30%O_2, 70%O_2)	530.4	C-O	ether, hydroxyl[9]
		531.3	C=O	Quinone[10,11]
		532.3	C-O-C	ether, hydroxyl[10]

Table S4. Fundamental data and final calculative results of capacity balance evaluation

	unsintered	100%N_2 sintered	O_2:N_2=3:7 sintered	O_2:N_2=7:3 sintered
	Capacity electrodes possessing (C/g)			
Prior nitrate reduction[1]	18.96	6.05	9.30	10.84
After nitrate reduction[2]	19.18	6.12	9.28	11.15
	Capacity equivalent of electrons transformed based on nitrate reduction (C/g)			
a: Based on NO_3^- loss	10.18	5.24	7.11	9.18
b: Based on NH_4^+ formation	12.75	3.77	3.67	4.43
Total (a+b)	22.93	9.01	10.78	13.61
CCR	119.6%	123.1%	116.2%	122.1%

[1,2] Shown were calculated based on current profiles in Fig. S11 according to the formula: $C = \int_0^T Itdt$.

Table S5. Evaluation of ORR catalytic capability of CB based electrodes with different pre-treatments

	Unsintered	100%N_2 sintered	O_2:N_2=3:7 sintered	O_2:N_2=7:3 Sintered
COD removal amount (mg/L)	907.9±15.8	842.19±10.8	799.8±18.6	1041.7±15.1
COD Removal Efficiency (%)	78.9%	60.5%	63.8%	68.7%
Capacity equivalent for current output (C)[a]	280.0	148.7	169.8	245.3
Coulombic Efficiency[b]	55.7%	38.5%	41.8%	56.0%

[a] Shown were calculated based on current profiles in Fig. 6 according to the formula: $C = \int_0^T Itdt$; [b] Ration of calculated capacity equivalent of current profile (Fig. 6) to capacity equivalent of COD removal.

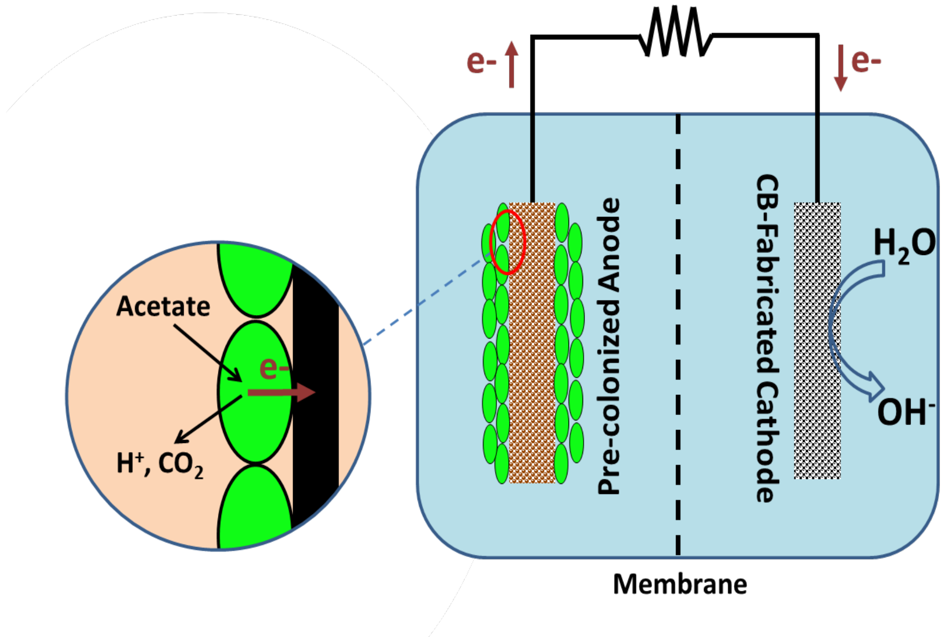

Fig. S1 The construction and schematic diagram of the system for bioelectrochemical measurement

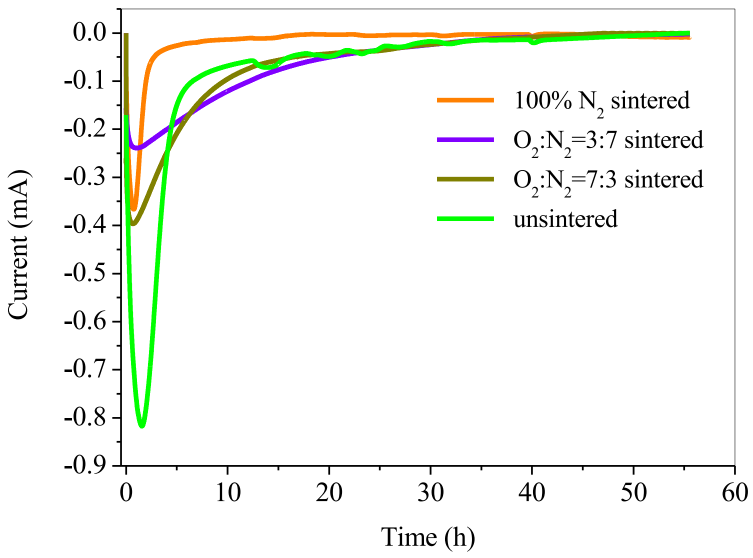

Fig. S2 Current curves of charging differently treated CB electrodes at the potential of -1.6 V

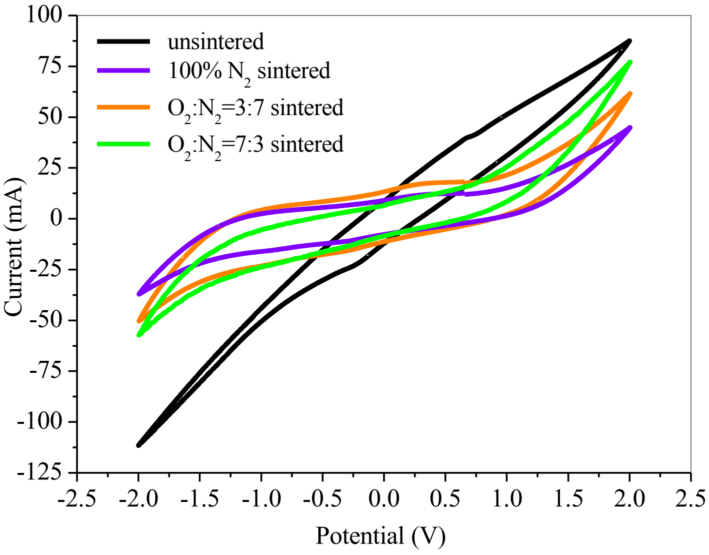

Fig. S3 The cyclic voltammogram curves of charged CB-based electrodes

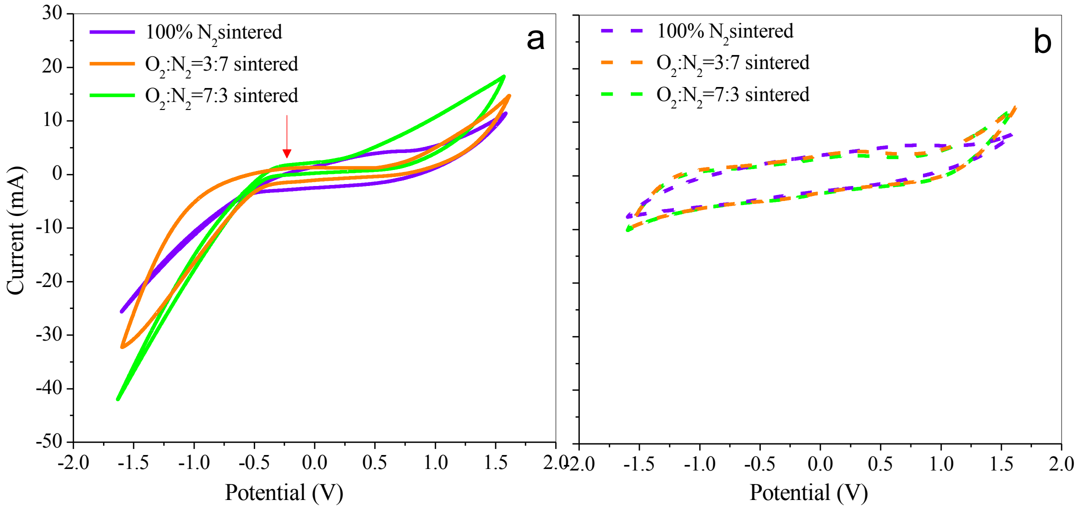

Fig S4 The cyclic voltammogram curves of carbon black based electrodes (a: the curves of newly fabricated electrodes with different pre-treatments; b: corresponding curves of charged (fully saturated with electrons) electrodes)

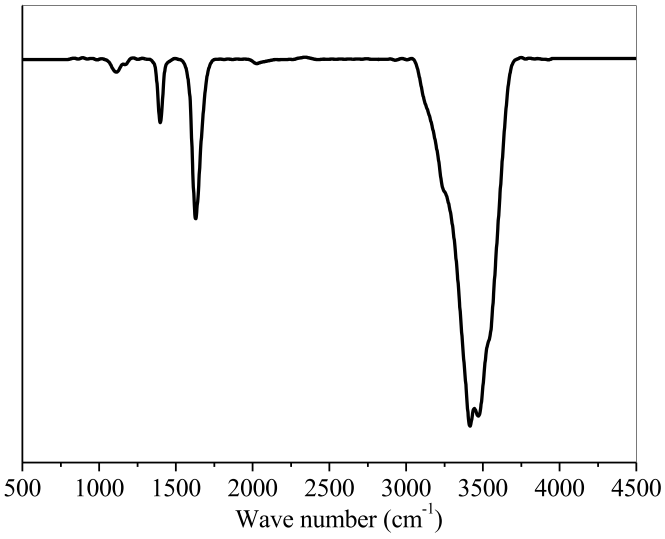

Fig. S5 FTIR profiles of CB powder

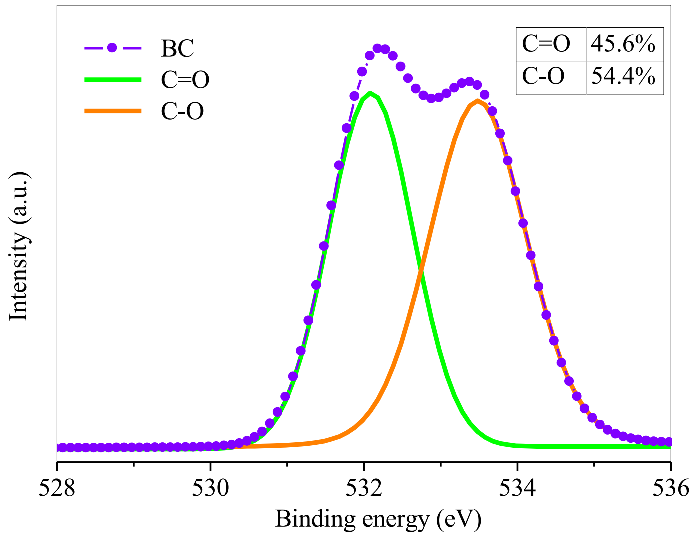

Fig. S6 O1 XPS profile and fitting peaks of CB powder

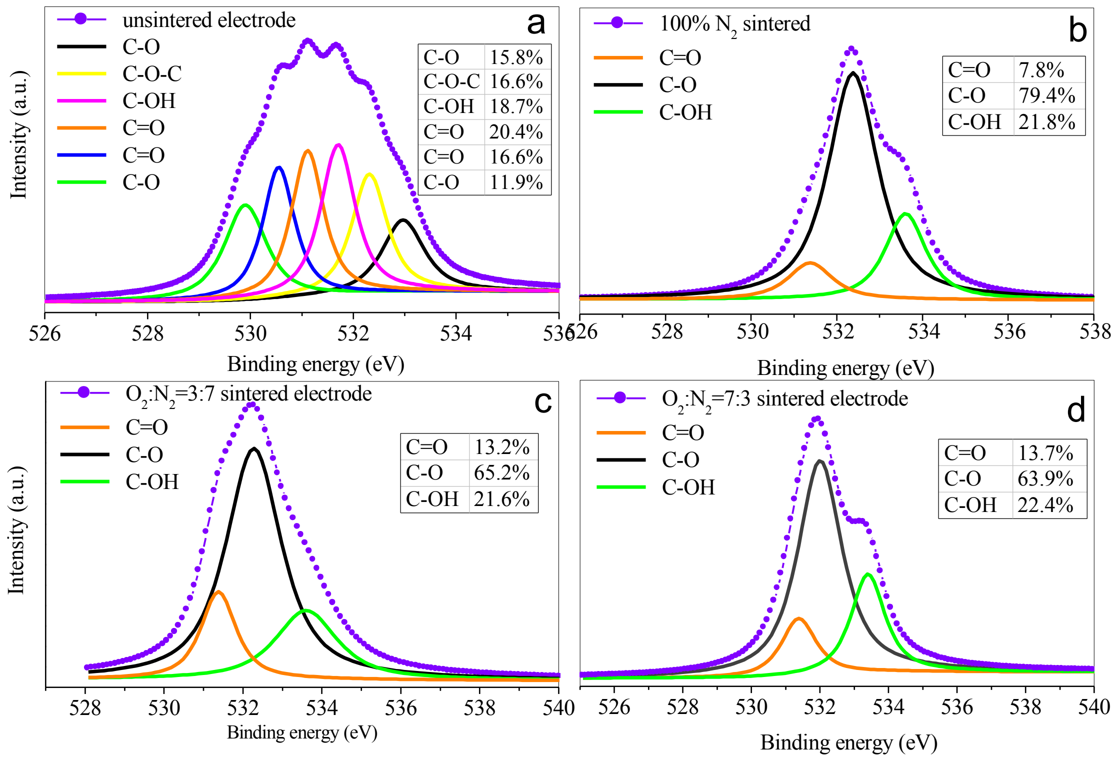

Fig. S7 The high resolution O1s spectra of charged CB-based electrodes with different pre-treatments

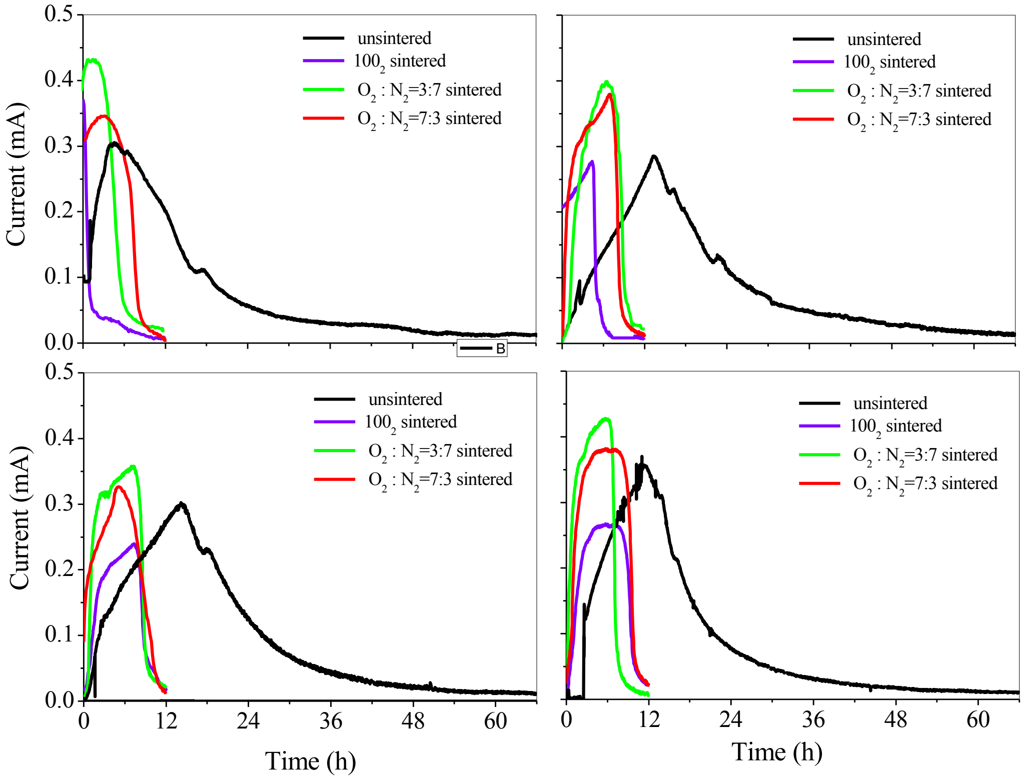

Fig. S8 Charging curves of CB-based electrodes (a: the newly fabricated electrodes with different pre-treatments; b: charged electrodes in 'a' discharged with 0.1 mA constant current; c: charged electrodes in 'a' discharged with 0.5 mA constant current; d: charged electrodes in 'a' discharged with 1.0 mA constant current)

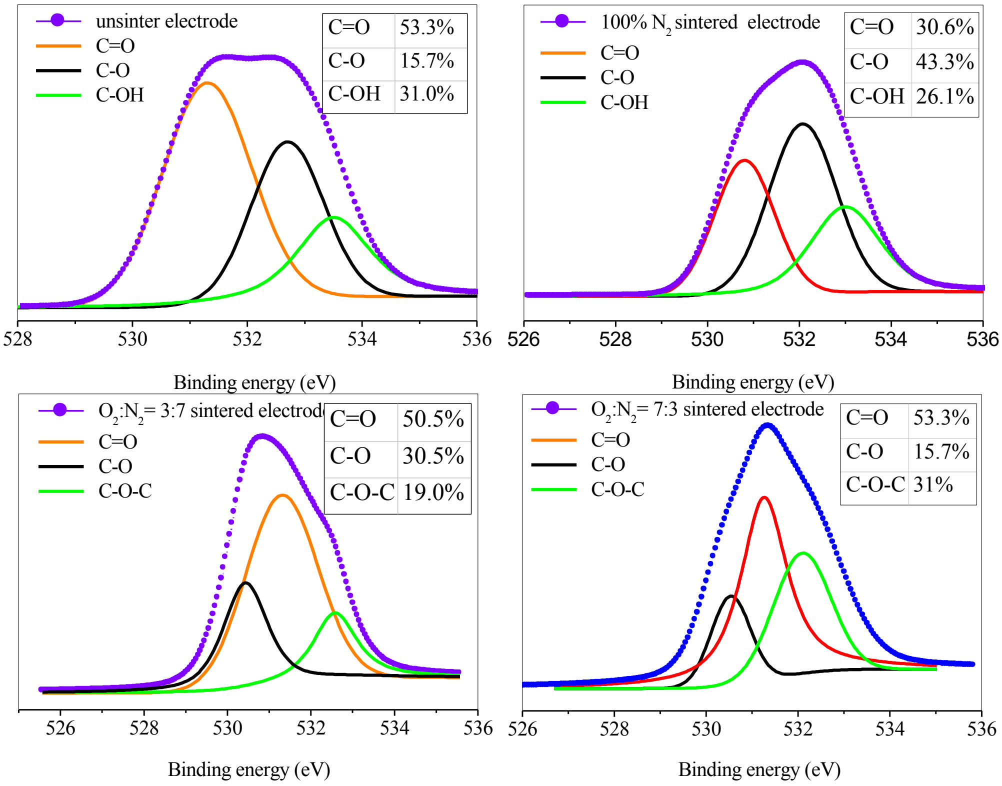

Fig. S9 The high resolution O1s spectra of discharged CB-based electrodes with different pre-treatment

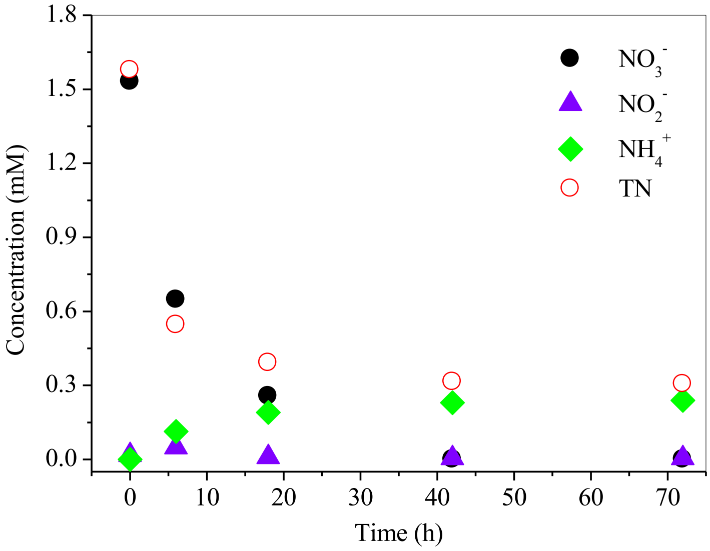

Fig. S10 Nitrate metabolism kinetics of enriched nitrate-reduction bacteria with H_2 as electron donor on day 30

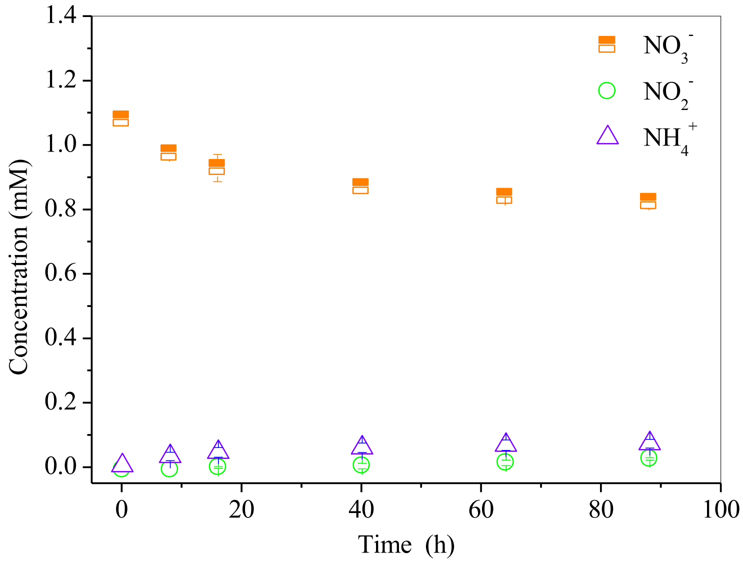

Fig. S11 Nitrate metabolism kinetics of microbial control without CB-based electrodes or H_2 as electron donors

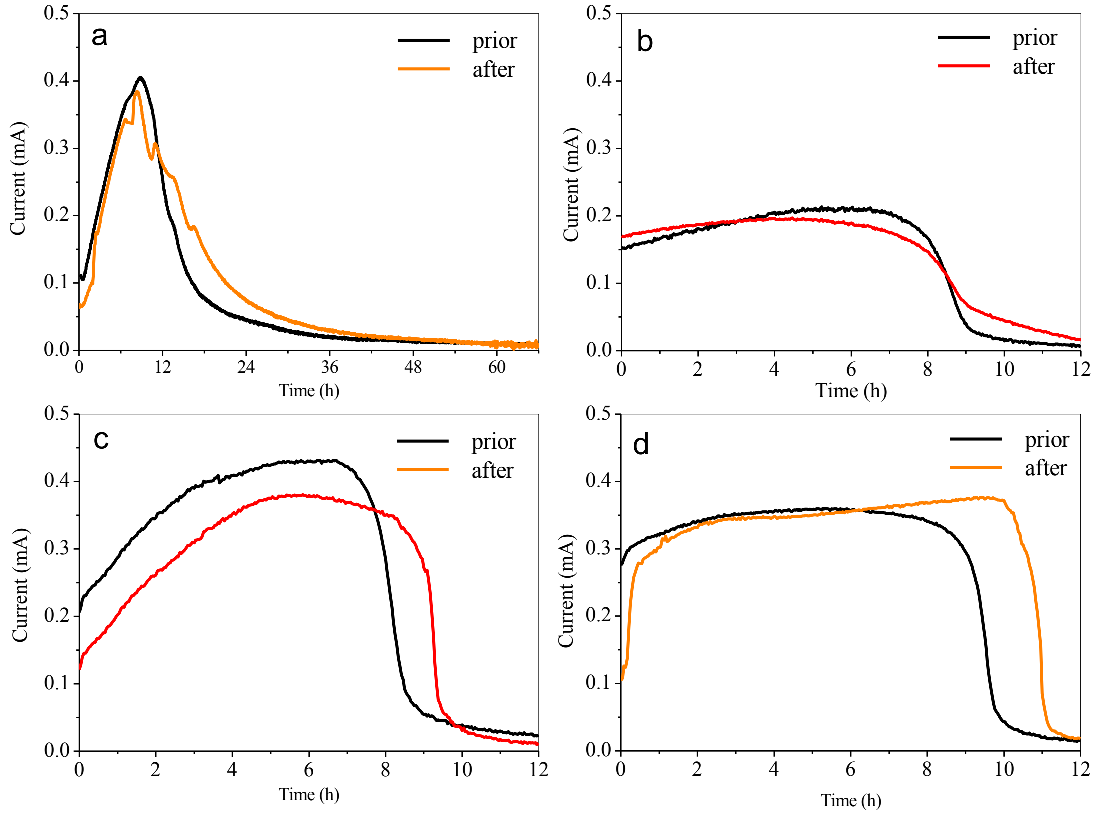

Fig. S12 Charging curves of the same CB-based electrodes prior and after nitrate reduction (a: electrode unsintered; b: electrodes sintered under 100% N_2; c: electrodes sintered under 30% O_2 and 70% N_2; d: electrodes sintered under 70% O_2 and 30% N_2)

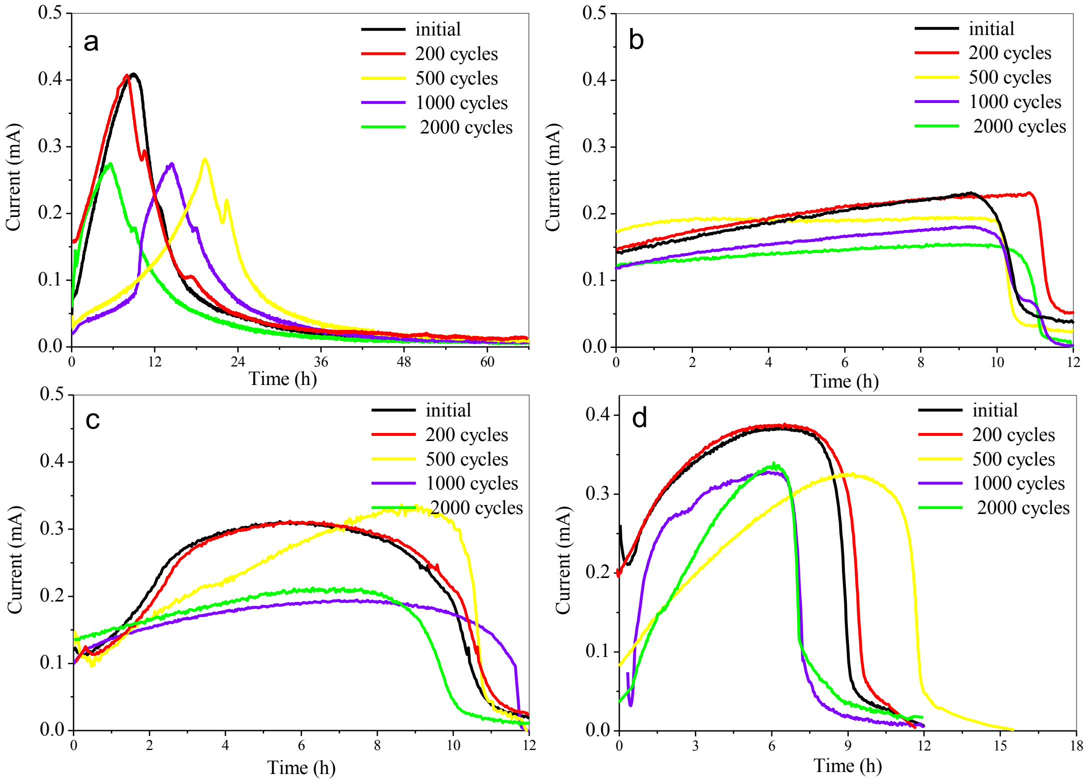

Fig. S13 Charging curves of the CB-based electrodes with different charging-discharging cycling (a: electrode unsintered; b: electrodes sintered under 100% N_2; c: electrodes sintered under 30% O_2 and 70% N_2; d: electrodes sintered under 70% O_2 and 30% N_2)

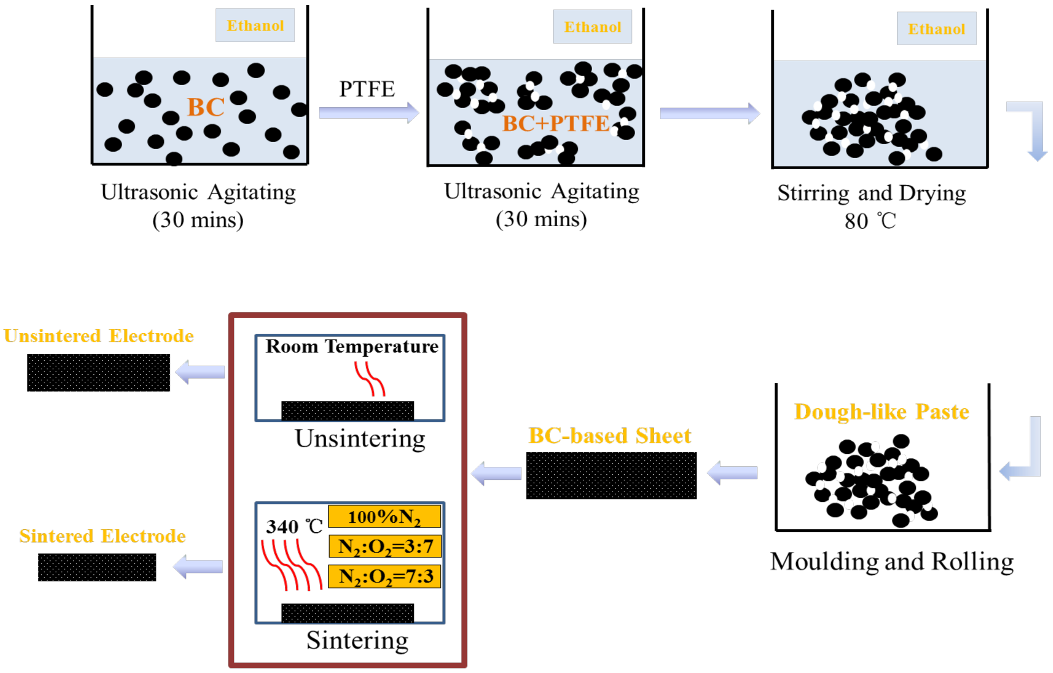

Fig. S14 Schematic diagram of procedures fabricating CB into electrodes

Reference

1. Bustin, R. & Guo, Y. Abrupt changes (jumps) in reflectance values and chemical compositions of artificial charcoals and inertinite in coals. *Int. J. Coal Geol.* **38**, 237−260 (1999).
2. Haberhauer, G., Rafferty, B., Strebl, F. & Gerzabek, M. Comparison of the composition of forest soil litter derived from three different sites at various decompositional stages using FTIR spectroscopy. *Geoderma* **83**, 331−342 (1998).
3. Chen, B., Zhou, D. & Zhu, L. Transitional adsorption and partition of nonpolar and polar aromatic contaminants by biochars of pine needles with different pyrolytic temperatures. *Environ. Sci. Technol.* **42**, 5137−5143 (2008).
4. Kumar, A. et al. Combined experimental (FT-IR, UV–visible spectra, NMR) and theoretical studies on the molecular structure, vibrational spectra, HOMO, LUMO, MESP surfaces, reactivity descriptor and molecular docking of Phomarin. *J Mol. Struct.* **1096**, 94–101 (2015).
5. López-Pasquali, C. E. & Herrera, H. Pyrolysis of lignin and IR analysis of residues. *Thermochim. Acta* **293**, 39-46 (1997).
6. Pradhan, B. & Sandle, N. Effect of different oxidizing agent treatments on the surface properties of activated carbons. *Carbon* **37**, 1323-1332 (1999).
7. Pretsch, E., Bühlmann, P. & Badertscher, M. *Structure Determination of Organic Compounds*. Springer-Verlag: Berlin, 2009.
8. Smith, D. M. & Chughtai, A. R. The surface-structure and reactivity of black carbon. *Colloid Surface A* **105**, 47-77 (1995).
9. Valdes, H., Sanchez-Polo, M., Rivera-Utrilla, J. & Zaror, C.A. Effect of ozone treatment on surface properties of activated carbon. *Langmuir* **18**, 2111−2116 (2002).
10. Zielke, U., Huttinger, K. J. & Hoffman W. P. Surface-oxidized carbon fibers: I. Surface structure and chemistry. *Carbon* **34**, 983–998 (1996).
11. Pantea, D., Darmstadt, H., Kaliaguine, S. & Roy, C. Electrical conductivity of conductive carbon blacks: influence of surface chemistry and topology. *Appl.*

Surf. Sci. **217**, 181–193 (2003).

12. Marzorati, S., Ragg, E. M., Longhi, M., Formaro, L. Low-temperature intermediates to oxygen reduction reaction catalysts based on amine-modified metal-loaded carbons. An XPS and ss-NMR investigation. *Mater. Chem. Phys.* **162**, 234–243 (2015).
13. Strzemieckaa, B., Voelkela, A., Donate-Roblesb, J. & Martín-Martínezb, J. M. Assessment of the surface chemistry of carbon blacks by TGA-MS, XPS and inverse gas chromatography using statistical chemometricanalysis. *Appl. Surf. Sci.* **316**, 315–323 (2014).
14. Yan, J. et al. Template-assisted low temperature synthesis of functionalized graphene for ultrahigh volumetric performance supercapacitors. *Acsnano* **8**, 4720–4729 (2014).
15. Frackowiak, E. & Beguinb. F. Carbon materials for the electrochemical storage of energy in capacitors. *Carbon* **39**, 937–950 (2011).
16. Cory, R. M. & McKnight, D. M. Fluorescence spectroscopy reveals ubiquitous presence of oxidized and reduced quinones in dissolved organic matter. *Environ. Sci. Technol.* **39**, 8142–8149 (2005).
17. Scott, D. T., McKnight, D. M., Blunt-Harris, E. L., Kolesar, S. E. & Lovley, D. R. Quinone moieties act as electron acceptors in the reduction of humic substances by humics-reducing microorganisms. *Environ. Sci. Technol.* **32**, 2984–2989 (1998).
